# Fatigue in long-term cancer survivors: prevalence, associated factors, and mortality. A prospective population-based study

**DOI:** 10.1038/s41416-025-03116-z

**Published:** 2025-07-15

**Authors:** Melissa S. Y. Thong, Daniela Doege, Lena Koch-Gallenkamp, Heike Bertram, Andrea Eberle, Bernd Holleczek, Alice Nennecke, Annika Waldmann, Sylke Ruth Zeissig, Ron Pritzkuleit, Elmar Brähler, Hermann Brenner, Volker Arndt

**Affiliations:** 1 Cancer Survivorship, Division of Clinical Epidemiology and Aging Research, German Cancer Research Center (DKFZ), Heidelberg, Germany; 2 Division of Clinical Epidemiology and Aging Research, DKFZ, Heidelberg, Germany; 3 Cancer Registry of North Rhine-Westphalia, Bochum, Germany; 4 Bremen Cancer Registry, Leibniz Institute for Prevention Research and Epidemiology - BIPS, Bremen, Germany; 5 Saarland Cancer Registry, Saarbrücken, Germany; 6 Hamburg Cancer Registry, Hamburg, Germany; 7 Institute for Social Medicine and Epidemiology, University of Lübeck, Lübeck, Germany; 8 Institute of Clinical Epidemiology and Biometry (ICE-B), Julius Maximilian University of Würzburg, Würzburg, Germany; 9 Cancer Registry of Rhineland-Palatinate, Mainz, Germany; 10 Cancer Registry of Schleswig-Holstein, Lübeck, Germany; 11 Department of Medical Psychology and Medical Sociology, University Hospital Leipzig, Leipzig, Germany; 12 Department of Psychosomatic Medicine and Psychotherapy, University Hospital Medical Center of the Johannes Gutenberg University Mainz, Mainz, Germany; 13 German Cancer Consortium (DKTK), DKFZ, Heidelberg, Germany

**Keywords:** Fatigue, Cancer epidemiology

## Abstract

**Background:**

We compared fatigue severity in breast, prostate or colorectal cancer survivors 5–16 years post-diagnosis with cancer-free controls, and examined factors associated with fatigue and its association with all-cause mortality in survivors.

**Methods:**

Participants of the *CA*nc*E*r *S*urvivorship - *A* multi-*R*egional (CAESAR) study completed the Fatigue Assessment Questionnaire (FAQ) between 2009 and 2011. The FAQ assesses affective, cognitive, and physical fatigue, and sleep problems. We derived the odds of fatigue using logistic regression with the 75^th^ percentile of population norms as the cut-off. All-cause mortality (up to end 2021) was estimated using Cox regression models.

**Results:**

The sample comprised 6057 survivors, of whom approximately one-third reported affective, cognitive, or physical fatigue. Demographic (age, relationship), clinical (chemotherapy), comorbidity (depression), lifestyle, and psychological factors were associated with higher odds of fatigue symptoms and total fatigue. Fatigue symptoms, predominantly physical fatigue, were strongly associated with mortality (unadjusted hazard ratios (HRs) ranged from 1.48 to 2.40). The HRs were attenuated after adjustment for comorbidities and depressive symptoms, although affective and physical fatigue remained independent risk factors for mortality.

**Conclusions:**

Demographic, clinical, comorbidity, lifestyle, and psychological factors were associated with fatigue in long-term survivors. Fatigued survivors have a higher mortality risk. Lowering the burden of fatigue by a comprehensive approach might result in better survival.

## Introduction

Cancers of the breast, prostate, colon, and rectum are among the most common cancers in developed countries [1]. These cancers often have relatively favorable prognosis. Despite the fact that individuals are living longer after a cancer diagnosis, a considerable number of cancer survivors continue to experience adverse effects resulting from the disease and its treatment for many years after the end of active treatment [2].

Fatigue is a common symptom, often triggered by cancer and its treatment [3]. Up to 85% of survivors undergoing active treatment report feeling fatigued [4]. Although fatigue does subside after the completion of active treatment [5], between 20% to 60% of cancer survivors still feel fatigued long after treatment completion [6–8]. Approximately 34% of breast cancer survivors reported significant fatigue 5–10 years post-diagnosis [9]. Similarly, population-based studies that included long-term ( ≥ 5 years post-diagnosis) colorectal cancer survivors reported higher levels of fatigue among survivors than controls [6, 10]. On the other hand, there was no difference in fatigue levels between survivors of prostate cancer diagnosed 10-years previously and age-matched controls [11].

The etiology of fatigue is inconclusive. Fatigue is a complex and multifactorial symptom. In addition to active treatment, other factors have been identified to be associated with fatigue in long-term cancer survivors. These include being female, younger age, lower education level, treated with chemotherapy, being on maintenance therapy, psychological distress, or having comorbid conditions [6, 12–14].

Fatigue is generally considered as one phenomenon that can be experienced in different forms (‘multidimensional concept’), e.g., physical, mental, or emotional dimensions of fatigue. This concept assumes that the different dimensions of fatigue stem from the same cause, and thus do not need to be differentiated in its treatment [15]. However, it has been postulated that fatigue may be a collective term for distinct symptoms (‘multiple symptom’ concept) [15]. This is based on studies that have found different prevalence and correlates for mental/cognitive, emotional/affective, and physical fatigue [5, 16, 17]. Breast cancer survivors who have higher baseline body mass index and worries about the future, or had received chemotherapy treatment were more likely to experience physical fatigue [5]. Survivors who were obese or had poor social support were more likely to report having affective fatigue [5]. Baseline psychopharmaceutical use and poor sleep were associated with physical and cognitive fatigue [5]. According to the multiple symptom concept of fatigue, the different fatigue symptoms could have a different pathogenesis and require different treatments [15].

Besides being associated with greater disability and poorer health-related quality of life (HRQOL) [18, 19], fatigue is also associated with poorer survival among survivors of breast, prostate or colorectal cancer [20–25]. However, the results are equivocal and studies to date tended to focus on survivors within 5-year post-diagnosis. One study with a longer follow-up reported that breast cancer survivors who were fatigued after end of active treatment had increased risks of mortality during follow-up of 10 or more years [26, 27].

Studies exploring the affective, cognitive, and physical fatigue experiences of long-term cancer survivors on prognosis are limited. Published studies tended to focus on fatigue as a general concept or included specific groups of cancer survivors [5, 9, 11, 28], with short- and/or long-term survivorship [5, 6], or had small sample size [28]. A deeper insight into the potential different symptoms of fatigue in long-term survivors could improve the provision of survivorship care adapted to survivors’ needs. Our three main study objectives were: 1) to compare affective, cognitive, physical, and total fatigue in long-term (5–16 years post diagnosis) survivors of breast, prostate or colorectal cancer and age- and sex-matched non-cancer controls; 2) to investigate whether affective, cognitive, physical, and total fatigue are associated with demographic (age at survey, sex, education), clinical (cancer type, disease status, comorbidity), psychological, and lifestyle factors, and 3) to investigate the association of fatigue (affective, cognitive, physical, and total) with all-cause mortality in cancer survivors.

## Methods

### Setting and participants

#### Caesar

The population-based CAncEr Survivorship - A multi-Regional (CAESAR) study aimed to describe the long-term HRQOL of breast, colorectal, and prostate cancer survivors (Supplementary Fig. 1). The German Cancer Research Center (Deutsches Krebsforschungszentrum, DKFZ) conducted the study, in collaboration with six population-based cancer registries in Germany (Bremen, Hamburg, North Rhine-Westphalia, Rhineland-Palatinate, Saarland, and Schleswig-Holstein). Cancer survivors diagnosed between January 1994 and June 2004 as registered in the participating cancer registries, and aged between 20 and 75 years at diagnosis were eligible. Exceptions were the registries in Schleswig-Holstein and Rhineland-Palatinate which recruited survivors diagnosed from year 2000 onwards as these registries were established only in the late 1990s.

Data was collected between March 2008 and May 2011 by postal questionnaire. Depending on the cancer registry, the participants were contacted directly by the cancer registry (Hamburg, Saarland, Schleswig-Holstein) or via the treating/study physician (Bremen, North Rhine-Westphalia, Rhineland-Palatinate). Non-respondents received up to two reminders and a telephone contact.

The ethics committee of the University of Heidelberg (S499/2012) and the responsible local ethics committees of the participating cancer registries approved the study. All participants provided written informed consent.

#### Non-cancer (population) controls

A representative sample of the German population provided individual level fatigue scores. Participants aged 14–92 years (*n* = 2507) were selected from 210 sample points in Germany, and were assessed in their homes by trained interviewers [29]. Data was collected in 2003 by USUMA (LLC), an independent market research institute based in Berlin, Germany. Further details of the sample selection are reported elsewhere [29]. For the current study, we selected participants who were of comparable age (range: 34–89 years, *n* = 1953) to the CAESAR sample as controls (Supplementary Fig. 1).

### Assessments

#### Fatigue Assessment Questionnaire (FAQ)

The 20-item FAQ assesses physical (11 items), affective (5 items), and cognitive (3 items) domains of fatigue [30]. A single item assesses sleep problems at night. Item scores ranged from 0 ‘not at all’ to 3 ‘very much’. Item scores are summed to derive three subscale (physical, affective, and cognitive) scores, which are then summed with the sleep problem score to derive the total fatigue score. The subscale scores are ranged: 0–15 (affective), 0–9 (cognitive), 0-33 (physical), 0–3 (sleep problem), and 0-60 (total). We determined fatigue cut-off with the 75^th^ percentile score of the FAQ subscales in adult non-cancer controls aged 18–92 (*n* = 2417, Supplementary Fig. 1). We used cut-offs of: ≥4 for affective fatigue; ≥3 for cognitive fatigue; ≥12 for physical fatigue; ≥1 for sleep problems; ≥19 for total fatigue.

#### Geriatric Depression Scale (GDS)

The 15-item GDS is a validated and reliable screening tool for depression and items are answered with either a yes (1) or a no (0) [31]. Out of a maximum score of 15, a score of ≥5 indicates symptoms of depression [32].

#### Fear of Progression Questionnaire (FoP-Q-SF)

We used the validated 12-item FoP-Q-SF to assess the frequency of fear/worry of disease progression. Items are scored on a range of 1 (‘never’) to 5 (‘very often’). Moderate fear of progression is indicated with a score of ≥4 on at least 50% of items and a high fear of progression is indicated with a score of ≥4 on at least 75% of items [33].

#### Demographics and clinical data

The CAESAR questionnaire contained questions concerning sociodemographic factors and clinical history. Self-reported data included treatment received, disease progression since index cancer (recurrence, metastasis, new primary cancer), and comorbid conditions at the time of the survey. The comorbid conditions were grouped as ‘cardiovascular disease’ (stroke, myocardial infarction, angina pectoris, heart failure), ‘skeletal’ (arthrosis, osteoporosis), ‘inflammation’ (rheumatism, diabetes mellitus), ‘depression’ (depression), and ‘multiple’ (having conditions that span different groups) in the analyses. Cancer site was classified according to the International Classification of Diseases-10 codes. The date of diagnosis and the tumor stage were provided by the participating cancer registries. The cancer registries provided data on vital status up to 31 December 2021.

### Statistical analyses

All analyses were conducted with SAS (version 9.4 for Windows; SAS Institute Inc., Cary, NC).

To reduce possible bias due to missing data (generally <10%), we conducted 25 imputations using the Markov chain Monte Carlo method to impute the missing data before commencing the analyses [34].

We used Cochran-Mantel Haenszel tests to compare the sociodemographic characteristics of cancer survivors and population controls. The age and sex distribution of the population controls reflected a stratified sampling scheme [2], but were still significantly different from the cancer survivors (Table 1). To address this discrepancy, we used direct standardization by age and sex for further comparisons of sample characteristics, using weights derived from the age and sex distribution of cancer survivors as standard.

**Table 1:** Sociodemographic characteristics of cancer survivors and population controls.

	Cancer Survivors		Population controls			P_crude_ (Χ^2^)	P_adjusted_ (CMH)^a^
	n	%	n	%	%adj^a^		
**Total**	6057	100	1953	100			
**Age at survey**						**<0.0001**	-
<60 years	925	15	1140	58	15		
60–69 years	1787	30	458	24	30		
70–79 years	2734	45	274	14	45		
≥80 years	611	10	81	4	10		
**Mean age at survey (SD)**	69.0	(9.1)	55.6	(13.7)			
**Sex:** male	2899	48	871	45	48	**0.01**	-
**Education**						**<0.0001**	**<0.0001**
≤9 years	3222	53	1028	53	69		
10–11 years	1450	24	595	31	19		
≥12 years	1386	23	330	17	12		
**Relationship**						**<0.0001**	**<0.0001**
Single	261	4	210	11	4		
Married / In relationship	4496	74	1217	62	60		
Divorced	428	7	210	11	7		
Widowed	871	14	316	16	29		
**Employment at survey**						**<0.0001**	**<0.0001**
Full-/part-time	902	15	901	46	14		
(Early) retired / Unemployed	4324	71	873	45	79		
Other	831	14	179	9	8		
**Net monthly household income**						**<0.0001**	**<0.0001**
<1000 euros	1130	19	311	16	20		
1000–2999 euros	3842	63	1513	78	76		
≥3000 euros	1085	18	129	7	4		

^a^Rates of population controls standardized by the age and sex distribution of the cancer survivors cohort. Χ^2^: chi-squared tests; CMH: Cochran-Mantel-Haenszel tests. All results are based on 25 imputations of missing values. Numbers might not add up to total sample size due to rounding of multiple imputation results. Percentages might not add up to 100% due to rounding of percentages. Statistically significant *p*-values are highlighted in bold (*p* < 0.05).

We compared the least square mean FAQ subscale and total scores of survivors and controls with multiple linear regression. The regression models were adjusted for age at time of survey, sex, and education, where appropriate. These covariates were selected a priori, based on previous reported associations with fatigue [6, 13]. In models stratified by disease status, we categorized the survivors as follows: those diagnosed with stage I-III disease and with no self-reported recurrence, metastasis or new cancer at the time of survey were classified as ‘disease-free’. Those who were diagnosed with stage IV or with a self-reported recurrence, metastasis or new cancer after index cancer were classified as ‘active disease’. Two-sided statistical significance was determined with *p* < 0.05.

Using the FAQ cut-off scores, we derived the adjusted odds ratios (OR_adj_) and two-sided 95% confidence intervals (95% CI) of demographic, clinical, comorbidity, lifestyle, and psychological variables associated with fatigue among cancer survivors. These models were adjusted for age at survey, sex, and cancer stage, where appropriate.

Associations between fatigue (affective, cognitive, physical, sleep problems, total) and all-cause mortality were estimated with Cox proportional hazards models. Covariates, selected a priori, included for adjustment in the Cox models were age at survey, sex, education, years since diagnosis, cancer type, comorbidity, smoking, BMI, physical activity, and depressive symptoms [20, 21, 26]. Estimated survival duration was from time of study invitation until censoring, death or end of follow-up on 31 December 2021. We tested the proportional hazards assumption by checking the *p*-values of the time-dependent explanatory variables. To address the violation of the proportional hazards assumption when time of follow-up was modelled from time of survey to the end of 2021, we made an arbitrary split of the follow-up into 2 periods (short follow-up: up to 5 years since survey completion; longer follow-up: 6 years after survey completion to the end of follow-up). In addition, for covariates that still showed a violation of the proportional hazards assumption after stratification of the follow-up time, we introduced an interaction term between the covariate and time into the model [35]. As disease status and fatigue could be associated with mortality [22], we ran sensitivity analyses excluding survivors with active disease.

## Results

### Response analysis of cancer survivors

Of the 14,526 cancer survivors eligible for study participation, 6057 (41%) returned a complete questionnaire (Supplementary Fig. 1). Respondents were younger, had shorter time since diagnosis, and less likely to have had a diagnosis of colorectal cancer (data not shown).

### Characteristics of cancer survivors and population controls

Cancer survivors were more likely to be older (mean age: 69.0 ± 9.1 versus 55.6 ± 13.7, *p* < 0.0001) and male (48% versus 45%, *p* = 0.01) compared to controls (Table 1). Even after standardization of age and sex, significant differences remained. Cancer survivors were more likely to have ≥12 years of education, be in a partnered relationship, have a net monthly household income of ≥3000 euros, and less likely to be in early retirement or unemployment (all *p* < 0.0001).

### Prevalence of fatigue and mean fatigue scores of cancer survivors and population controls

Between 34 and 39% of cancer survivors were fatigued and 63% reported sleep problems, while about 28–33% of the population controls were fatigued and 47% reported sleep problems (Supplementary Table 1).

#### By age at survey

**Fig. 1:**
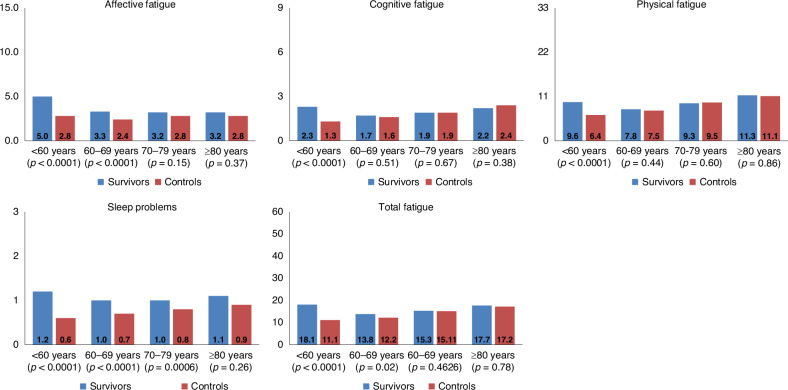
Mean fatigue scores of cancer survivors and non-cancer controls, stratified by age at survey. Models are adjusted for age at survey, gender, and education. The y-axis indicates the maximum score range and the mean values are shown in the columns. All results are based on 25 imputations of missing values.

The mean levels of all symptoms of fatigue and total fatigue were higher in cancer survivors when compared with controls, in particular among the younger age groups <60 years and 60-69 years (Fig. 1). Survivors in the age groups of <60, 60–69, and 70–79 years reported higher levels of sleep problems compared to controls.

#### By education level

Irrespective of education level, the mean levels of affective fatigue and sleep problems were significantly higher among cancer survivors when compared with controls (Supplementary Fig. 2). Survivors with fewer years of education (up to 11 years) reported higher levels of cognitive, physical, and total fatigue when compared with controls of same education pedigree.

#### By sex and cancer type

**Fig. 2:**
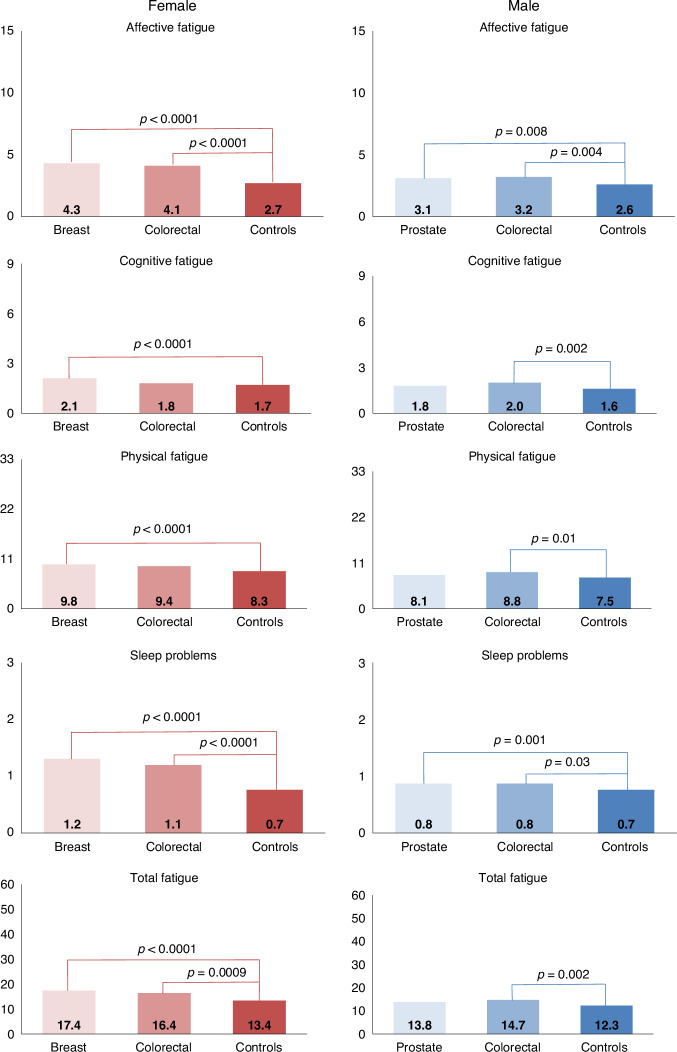
Mean fatigue scores of cancer survivors and non-cancer controls, stratified by sex and by cancer type. Models are adjusted for age at survey and education. The *p*-values indicate the global comparison between cancer survivors and controls, separate by sex. The y-axis indicates the maximum score range and the mean values are shown in the columns. All results are based on 25 imputations of missing values.

Female breast cancer survivors reported significantly higher scores in all symptoms of fatigue, total fatigue, and in sleep problems than female controls (Fig. 2). In comparison with controls, female colorectal cancer survivors reported higher levels of affective and total fatigue, and more sleep problems.

Significant differences between prostate cancer survivors and controls were observed in affective fatigue and sleep problems. Male colorectal cancer survivors scored significantly higher on all symptoms of fatigue, total fatigue, and sleep problems when compared with male controls.

#### By disease status

Regardless of disease status, cancer survivors reported significantly higher levels on all symptoms of fatigue, total fatigue, and sleep problems, when compared with controls (Supplementary Fig. 3). However, the greatest difference in scores were noted between survivors with active disease and controls for physical and total fatigue.

#### By employment status

Cancer survivors, irrespective of employment status, reported higher means on affective and total fatigue, and sleep problems when compared with controls (Supplementary Fig. 4). On cognitive and physical fatigue, survivors who were in full/part-time employment reported higher mean scores than employed controls.

### Factors associated with fatigue in cancer survivors

#### Demographic factors

With reference to the 70–79 years age group, younger age ( < 60 years of age) was associated with higher odds for affective (OR_adj_: 1.88, 95% CI: 1.60–2.21), cognitive (OR_adj_: 1.31, 95% CI: 1.11–1.54), and total fatigue (OR_adj_: 1.22, 95% CI: 1.04–1.44) (Table 2). Survivors in the oldest age group ( ≥ 80 years) had higher odds for cognitive (OR_adj_: 1.32, 95% CI: 1.10–1.58), physical (OR_adj_: 1.66, 95% CI: 1.39–1.98), and total (OR_adj_: 1.44, 95% CI: 1.20-1.72) fatigue, and had more sleep problems (OR_adj_: 1.20, 95% CI: 1.00–1.44). In contrast, the age group 60–69 years had lower odds for physical (OR_adj_: 0.71, 95% CI: 0.62–0.81) and total (OR_adj_: 0.82, 95% CI: 0.72–0.94) fatigue. Being male was associated with 20–49% lower odds for affective, physical, and total fatigue, and sleep problems. Having higher levels of education ( ≥ 10 years) reduced the odds for all symptoms of fatigue, total fatigue, and for sleep problems by 16–46% when compared with survivors with ≤9 years of education. However, not being in a partnered relationship was associated with increased odds in all symptoms of fatigue and total fatigue by 13–16%. Compared with unemployment/early retirement, current full/part-time employment was associated with 23–42% lower odds for all symptoms of fatigue, sleep problems, and total fatigue.

**Table 2:** Characteristics of cancer survivors and associations with fatigue.

Total (n = 6057)	Fatigued cancer survivors										
		Affective fatigue (overall prevalence 39%)		Cognitive fatigue(overall prevalence 35%)		Physical fatigue (overall prevalence 34%)		Sleep problems (overall prevalence 63%)		Total fatigue (overall prevalence 35%)	
	%col	Prev (%)	OR_adjusted_* (95% CI)	Prev (%)	OR_adjusted_* (95% CI)	Prev (%)	OR_adjusted_* (95% CI)	Prev (%)	OR_adjusted_* (95% CI)	Prev (%)	OR_adjusted_* (95% CI)
Demographic factors											
**Age at survey**											
<60 years	15	55	**1.88 (1.60**–**2.21)**	40	**1.31 (1.11**–**1.54)**	36	0.96 (0.81–1.13)	68	1.05 (0.89–1.25)	42	**1.22 (1.04**–**1.44)**
60–69 years	30	39	1.10 (0.97–1.25)	32	0.89 (0.78–1.01)	28	**0.71 (0.62**–**0.81)**	65	1.11 (0.98–1.26)	31	**0.82 (0.72**–**0.94)**
70–79 years	45	35	Ref.	34	Ref.	34	Ref.	59	Ref.	34	Ref.
≥80 years	10	35	1.05 (0.88–1.27)	41	**1.32 (1.10**–**1.58)**	46	**1.66 (1.39**–**1.98)**	63	**1.20 (1.00**–**1.44)**	42	**1.44 (1.20**–**1.72)**
**Sex**											
Female	52	45	Ref.	35	Ref.	35	Ref.	70	Ref.	38	Ref.
Male	48	32	**0.67 (0.60**–**0.75)**	35	1.05 (0.94–1.18)	32	**0.80 (0.71**–**0.90)**	54	**0.51 (0.45**–**0.57)**	32	**0.75 (0.67**–**0.85)**
**Education**											
≤9 years	53	41	Ref.	39	Ref.	39	Ref.	65	Ref.	40	Ref.
10–11 years	24	38	**0.77 (0.67**–**0.87)**	33	**0.77 (0.67**–**0.88)**	31	**0.71 (0.62**–**0.81)**	63	**0.84 (0.74**–**0.96)**	33	**0.69 (0.61**–**0.79)**
≥12 years	23	35	**0.73 (0.64**–**0.84)**	29	**0.60 (0.52**–**0.69)**	25	**0.54 (0.46**–**0.62)**	57	**0.77 (0.67**–**0.88)**	28	**0.57 (0.50**–**0.66)**
**In a partnered relationship**											
No	26	43	**1.13 (1.00**–**1.27)**	37	**1.13 (1.00**–**1.28)**	38	**1.16 (1.02**–**1.31)**	65	0.92 (0.81–1.05)	39	**1.14 (1.01**–**1.29)**
Yes	74	38	Ref.	34	Ref.	32	Ref.	62	Ref.	34	Ref.
**Employed**											
Yes	15	44	**0.64 (0.53**–**0.79)**	34	**0.77 (0.63**–**0.94)**	28	**0.58 (0.47**–**0.72)**	62	**0.68 (0.55**–**0.82)**	33	**0.62 (0.51**–**0.77)**
No	71	37	Ref.	35	Ref.	34	Ref.	61	Ref.	35	Ref.
Other	14	46	1.01 (0.85–1.19)	37	1.08 (0.92–1.28)	39	1.09 (0.92–1.29)	72	1.12 (0.93–1.34)	42	1.12 (0.95–1.32)
Clinical factors											
**Cancer type**											
Breast	44	46	Ref.	44	Ref.	35	Ref.	71	Ref.	38	Ref.
Colorectal	20	38	0.91 (0.75–1.11)	35	0.82 (0.67–1.01)	35	1.00 (0.82–1.23)	59	**0.75 (0.61**–**0.92)**	36	0.92 (0.75–1.13)
Prostate	36	32	0.89 (0.68–1.17)	35	**0.71 (0.54**–**0.94)**	31	0.85 (0.64–1.11)	55	0.79 (0.60–1.03)	31	0.78 (0.59–1.02)
**Sex by cancer type**											
*Female*											
Breast	44	46	Ref.	35	Ref.	35	Ref.	71	Ref.	38	Ref.
CRC	8	43	0.96 (0.79–1.17)	31	0.84 (0.68-1.03)	37	0.99 (0.81-1.21)	65	**0.74 (0.60-0.91)**	37	0.91 (0.74-1.11)
*Male*											
CRC	12	35	1.05 (0.87-1.26)	38	1.17 (0.98-1.40)	34	1.16 (0.97-1.39)	54	0.93 (0.78-1.10)	35	1.17 (0.97-1.40)
Prostate	36	32	Ref.	35	Ref.	31	Ref.	55	Ref.	31	Ref.
**Time since diagnosis**											
5-9 years	80	39	1.00 (0.87-1.14)	35	0.93 (0.82-1.07)	33	1.03 (0.90-1.18)	62	0.97 (0.84-1.11)	35	1.03 (0.90-1.18)
≥10 years	20	39	Ref.	37	Ref.	35	Ref.	64	Ref.	36	Ref.
Chemotherapy**											
No	61	36	Ref.	34	Ref.	33	Ref.	60	Ref.	34	Ref.
Yes	39	44	1.06 (0.93-1.20)	37	**1.21 (1.07-1.38)**	35	1.13 (0.99-1.28)	67	1.02 (0.90-1.16)	38	1.10 (0.97-1.25)
Radiotherapy**											
No	43	36	Ref.	35	Ref.	33	Ref.	58	Ref.	34	Ref.
Yes	57	42	1.03 (0.92-1.15)	35	1.07 (0.96-1.20)	34	1.01 (0.89-1.13)	66	**1.14 (1.01-1.27)**	36	1.02 (0.90-1.14)
Hormone therapy**,^**a**^											
No	61	38	Ref.	35	Ref.	32	Ref.	62	Ref.	34	Ref.
Yes	39	42	1.01 (0.88-1.15)	34	0.92 (0.80-1.05)	35	1.10 (0.96-1.26)	69	**1.15 (1.00-1.32)**	37	1.05 (0.92-1.20)
**Disease status**											
Disease-free	90	38	Ref.	34	Ref.	33	Ref.	63	Ref.	34	Ref.
Active disease	10	45	**1.43 (1.20-1.70)**	42	**1.41 (1.19-1.69)**	45	**1.77 (1.48-2.11)**	65	**1.21 (1.01-1.45)**	44	**1.61 (1.35-1.92)**
Comorbidity											
**Self-reported comorbidity**^**b**^											
None	38	27	Ref.	24	Ref.	19	Ref.	56	Ref.	21	Ref.
CVD	8	28	**1.29 (1.04-1.61)**	36	**1.76 (1.43-2.17)**	30	**1.82 (1.46-2.27)**	54	1.11 (0.91-1.35)	29	**1.69 (1.36-2.11)**
Inflammation	5	31	**1.42 (1.09-1.84)**	32	**1.50 (1.16-1.95)**	30	**1.84 (1.41-2.40)**	53	0.98 (0.77-1.25)	29	**1.68 (1.29-2.20)**
Skeletal	16	32	**1.24 (1.05**–**1.46)**	28	**1.28 (1.08**–**1.52)**	29	**1.70 (1.42**–**2.03)**	65	**1.29 (1.10**–**1.51)**	30	**1.64 (1.38**–**1.95)**
Depression (ever)	6	69	**4.87 (3.81**–**6.23)**	50	**2.96 (2.35**–**3.73)**	47	**3.61 (2.85**–**4.58)**	74	**2.02 (1.56**–**2.60)**	55	**4.23 (3.35**–**5.35)**
Multiple	26	59	**4.25 (3.69**–**4.90)**	53	**3.62 (3.15**–**4.17)**	57	**5.55 (4.79**–**6.42)**	73	**2.14 (1.86**–**2.47)**	59	**5.47 (4.73**–**6.32)**
Lifestyle											
**Body Mass Index (kg/m**^**2**^**)**											
<25	32	40	Ref.	32	Ref.	30	Ref.	64	Ref.	32	Ref.
25– <30	47	38	1.06 (0.94–1.20)	36	**1.24 (1.09**–**1.40)**	33	**1.23 (1.08**–**1.40)**	62	1.06 (0.94–1.20)	35	**1.23 (1.08**–**1.39)**
≥30	21	41	**1.22 (1.05**–**1.41)**	38	**1.38 (1.18**–**1.60)**	42	**1.85 (1.59**–**2.15)**	62	1.06 (0.91–1.23)	42	**1.67 (1.44**–**1.94)**
**Smoking**											
Never	79	37	Ref.	34	Ref.	33	Ref.	62	Ref.	34	Ref.
Former	11	45	**1.32 (1.11**–**1.56)**	41	**1.34 (1.13**–**1.59)**	36	**1.22 (1.03**–**1.45)**	65	1.11 (0.93–1.32)	41	**1.36 (1.15**–**1.62)**
Current	10	49	**1.39 (1.17**–**1.66)**	38	**1.20 (1.00**–**1.43)**	39	**1.38 (1.16**–**1.66)**	64	0.96 (0.80–1.15)	43	**1.46 (1.22**–**1.74)**
**Moderate-to-vigorous physical activity 150** **minutes/ week**											
No	58	42	**1.45 (1.30**–**1.61)**	38	**1.38 (1.24**–**1.54)**	39	**1.78 (1.59**–**1.99)**	64	1.08 (0.97–1.20)	41	**1.69 (1.51**–**1.89)**
Yes	42	34	Ref.	31	Ref.	26	Ref.	61	Ref.	28	Ref.
Psychological											
**Geriatric Depression Scale**											
Asymptomatic	75	26	Ref.	24	Ref.	20	Ref.	57	Ref.	21	Ref.
Symptomatic	25	80	**11.83 (10.22**–**13.69)**	69	**7.14 (6.27**–**8.13)**	74	**11.16 (9.72**–**12.81)**	81	**3.15 (2.72**–**3.63)**	80	**14.68 (12.69**–**16.98)**
**Fear of progression**											
No	87	33	Ref.	30	Ref.	28	Ref.	60	Ref.	29	Ref.
Yes	13	82	**8.62 (7.12**–**10.45)**	66	**4.63 (3.94**–**5.45)**	75	**8.32 (6.97**–**9.92)**	84	**3.27 (2.67**–**4.00)**	79	**9.20 (7.66**–**11.06)**

Results are based on 25 imputations of missing values.

*Col* column, *Prev* prevalence, *OR* odds ratio, *CI* confidence interval.

The bold values indicate significant results.

*Models are adjusted for age at survey and sex, where appropriate; **Treatment models are adjusted for age at survey, sex, and cancer stage, ^a^Only breast and prostate cancer survivors reported hormone therapy use.

^b^Comorbidity grouped as: cardiovascular disease (CVD; stroke, myocardial infarction, angina pectoris, heart failure); inflammation (rheumatism, diabetes mellitus); skeletal (arthrosis, osteoporosis); multiple (having conditions that span different groups e.g., CVD and depression).

### Clinical factors

Colorectal cancer survivors (OR_adj_: 0.75, 95% CI: 0.61–0.92), and in particular, female colorectal cancer survivors (OR_adj_: 0.74, 95% CI: 0.60–0.91) were less likely to report sleep problems, in comparison with female breast cancer survivors. Prostate cancer survivors were less likely to report cognitive fatigue (OR_adj_: 0.71, 95% CI: 0.54–0.94). Chemotherapy was associated with 21% higher odds for cognitive fatigue. Radiotherapy and hormone therapy were associated with higher odds of having sleep problems by 14% and 15%, respectively. Having active disease at the time of survey was associated with higher odds of 41–77% for all symptoms of fatigue and total fatigue, and sleep problems (OR_adj_: 1.21, 95% CI: 1.01–1.45).

### Comorbidity

Having any comorbid conditions was associated with higher odds for all symptoms of fatigue and total fatigue than having no comorbidity (Table 2), with the highest odds noted for physical fatigue (cardiovascular comorbidity: OR_adj_: 1.82, 95% CI: 1.46–2.27; inflammation comorbidity: OR_adj_: 1.84, 95% CI: 1.41–2.40; skeletal comorbidity: OR_adj_: 1.70, 95% CI: 1.42–2.03). Having (ever had) depression was associated with higher odds for all symptoms of fatigue, total fatigue, and sleep problems than other comorbid conditions. The highest odds were observed for affective fatigue (OR_adj_: 4.87, 95% CI: 3.81–6.23) and total fatigue (OR_adj_: 4.23, 95% CI: 3.35–5.35). Having multiple morbid conditions was significantly associated with increased odds of fatigue, most notably in physical fatigue (OR_adj_: 5.55, 95% CI: 4.79–6.42).

### Lifestyle factors

Compared to a body mass index (BMI) of <25, a higher BMI was associated with higher odds of fatigue symptoms by 22–85%, with the highest odds ratio observed for BMI ≥ 30 for physical fatigue (OR_adj_: 1.85, 95% CI: 1.59–2.15). A history of smoking (former and current) was associated with higher odds for all symptoms of fatigue and total fatigue, in particular for current smokers with observed odds for affective (OR_adj_: 1.39, 95% CI: 1.17–1.66), cognitive (OR_adj_: 1.20, 95% CI: 1.00–1.43), physical (OR_adj_: 1.38, 95% CI: 1.16–1.66), and total (OR_adj_: 1.46, 95% CI: 1.22–1.74) fatigue than non-smokers. Not having at least 150 minutes per week of moderate-to-vigorous physical activity was associated with increased odds in all fatigue symptoms and total fatigue; affective (OR_adj_: 1.45, 95% CI: 1.30–1.61), cognitive (OR_adj_: 1.38, 95% CI: 1.24–1.54), physical (OR_adj_: 1.78, 95% CI: 1.59–1.99), and total (OR_adj_: 1.69, 95% CI: 1.51–1.89).

### Psychological factors

Being symptomatic on the GDS was associated with significantly increased odds for being fatigued, with highest odds noted for affective (OR_adj_: 11.83, 95% CI: 10.22–13.69), physical (OR_adj_: 11.16, 95% CI: 9.72–12.81), and total (OR_adj_: 14.68, 95% CI: 12.69–16.98) fatigue. Similarly, having fear of progression was associated with being fatigued, namely for affective (OR_adj_: 8.62, 95% CI: 7.12–10.45), physical (OR_adj_: 8.32, 95% CI: 6.97–9.92), and total (OR_adj_: 9.20, 95% CI: 7.66-11.06) fatigue. Symptoms of depression (OR_adj_: 3.15, 95% CI: 2.72–3.63) and fear of progression (OR_adj_: 3.27, 95% CI: 2.67-4.00) were associated with sleep problems.

### Fatigue and the risk of all-cause mortality

#### Short follow-up (up to 5 years after survey)

In the short follow-up period, the median follow-up time was 5.0 years. In total, there were 817 deaths during this period. The characteristics of those who died are shown in Supplementary Table 2. In the unadjusted model, all fatigue symptoms were associated with an increased mortality risk, with the highest hazard ratio (HR_unadj_) noted for physical (HR_unadj_: 2.40, 95% CI: 2.09–2.75) and total (HR_unadj_: 2.06, 95% CI: 1.80–2.37) fatigue (Table 3, Model 1). After adjusting for demographic factors, the mortality risk associated with affective fatigue increased from HR_unadj_: 1.49, 95% CI: 1.30–1.71 to adjusted HR (HR_adj_): 1.71, 95% CI: 1.48–1.96 (Model 2), while the risk associated with physical fatigue decreased from HR_unadj_: 2.40, 95% CI: 2.09–2.75 to HR_adj_: 2.23, 95% CI: 1.94–2.56. The adjusted risks for the other fatigue symptoms remained similar to the unadjusted values. There were no changes to the risk estimates for all fatigue symptoms following adjustments of clinical covariates (Model 3). Slight reductions of between 13% and 19% in mortality risks were observed after adjustment for comorbidity and lifestyle factors (Models 4 and 5). However, the largest attenuation of risk was noted after adjustment of psychological factors in the full model (Model 6). Nevertheless, affective (HR_adj_: 1.22, 95% CI: 1.03–1.43), physical (HR_adj_: 1.69, 95% CI: 1.44–2.00), and total (HR_adj_: 1.48, 95% CI: 1.25–1.76) fatigue remained as significant risk factors in the full model.

**Table 3:** Risk estimates of fatigue on mortality in cancer survivors – short follow-up (up to 5 years after survey).

	Total Deaths n = 817	Crude mortality rate (100/person- years)	Hazard ratios with 95% confidence interval					
			Unadjusted	Adjusted				
			Model 1	Model 2	Model 3	Model 4	Model 5	Model 6
**Affective Fatigue**								
No	426	2.47	ref	ref	ref	ref	ref	ref
Yes	391	3.67	**1.49 (1.30**–**1.71)**	**1.71 (1.48**–**1.96)**	**1.71 (1.49**–**1.96)**	**1.63 (1.41**–**1.88)**	**1.56 (1.35**–**1.81)**	**1.18 (1.00**–**1.40)**
**Cognitive Fatigue**								
No	461	2.51	ref	ref	ref	ref	ref	ref
Yes	356	3.72	**1.48 (1.29**–**1.70)**	**1.41 (1.22**–**1.62)**	**1.41 (1.23**–**1.62)**	**1.31 (1.13**–**1.51)**	**1.28 (1.11**–**1.48)**	0.98 (0.83–1.14)
**Physical fatigue**								
No	385	2.03	ref	ref	ref	ref	ref	ref
Yes	432	4.83	**2.40 (2.09**–**2.75)**	**2.23 (1.94**–**2.56)**	**2.23 (1.94-2.56)**	**2.14 (1.85**–**2.48)**	**2.04 (1.76**–**2.36)**	**1.66 (1.40**–**1.96)**
**Sleep problems**								
No	283	2.70	ref	ref	ref	ref	ref	ref
Yes	534	3.06	1.13 (0.98–1.31)	**1.23 (1.06**–**1.42)**	**1.23 (1.06**–**1.42)**	**1.19 (1.02**–**1.37)**	**1.19 (1.02**–**1.38)**	1.03 (0.89–1.20)
**Total fatigue**								
No	398	2.16	ref	ref	ref	ref	ref	ref
Yes	419	4.43	**2.06 (1.80**–**2.37)**	**2.05 (1.78**–**2.35)**	**2.05 (1.78**–**2.35)**	**1.96 (1.69**–**2.27)**	**1.86 (1.60**–**2.15)**	**1.45 (1.22**–**1.72)**

Model 2: adjusted for demographic factors (age at survey, sex, education).

Model 3: model 2 + clinical factors (years since diagnosis, cancer type).

Model 4: model 3 + comorbidity.

Model 5: model 4 + lifestyle factors (body mass index, smoking, physical activity).

Model 6: model 5 + psychological factors (depressive symptoms, fear of progression).

*HR* hazard ratio, *CI* confidence interval.

The bold values indicate significant results.

#### Longer follow-up (6 years or more after survey)

In the longer period of follow-up, the median follow-up time was 5.6 years and there were a total of 1198 deaths (Table 4, Supplementary Table 2). Although the various symptoms of fatigue were associated with a higher risk of mortality in the univariate models, these risks were lower than those observed in the shorter period of follow-up. After full adjustment of covariates, the risks associated with affective and physical fatigue became insignificant (Model 6). Of note, cognitive fatigue was a significant risk factor in the univariate model (HR_unadj_: 1.46, 95% CI: 1.28–1.66) and remained significant even after full adjustment (HR_adj_: 1.20, 95% CI: 1.05–1.38). Similarly, total fatigue (HR_adj_: 1.24, 95% CI: 1.04–1.41) remained significantly associated with mortality in the fully adjusted model.

**Table 4:** Risk estimates of fatigue on mortality in cancer survivors – longer follow-up (6 years or more after survey).

	Total Deaths n = 1198	Crude mortality rate (100/person- years)	Hazard ratios with 95% confidence interval					
			Unadjusted	Adjusted				
			Model 1	Model 2	Model 3	Model 4	Model 5	Model 6
**Affective Fatigue**								
No	751	4.43	ref	ref	ref	ref	ref	ref
Yes	447	4.50	1.11 (0.95–1.30)	**1.32 (1.13**–**1.55)**	**1.32 (1.13**–**1.55)**	**1.24 (1.06**–**1.45)**	**1.21 (1.03**–**1.42)**	1.02 (0.85–1.22)
**Cognitive Fatigue**								
No	714	3.94	ref	ref	ref	ref	ref	ref
Yes	484	5.53	**1.46 (1.28**–**1.66)**	**1.38 (1.22**–**1.57)**	**1.38 (1.21**–**1.57)**	**1.31 (1.15**–**1.49)**	**1.31 (1.15**–**1.50)**	**1.20 (1.05**–**1.38)**
**Physical fatigue**								
No	748	3.92	ref	ref	ref	ref	ref	ref
Yes	450	5.75	**1.60 (1.38**–**1.87)**	**1.49 (1.28**–**1.74)**	**1.49 (1.28**–**1.74)**	**1.39 (1.18**–**1.63)**	**1.33 (1.13**–**1.56)**	1.14 (0.96–1.36)
**Sleep problems**								
No	470	4.69	ref	ref	ref	ref	ref	ref
Yes	728	4.32	0.91 (0.81–1.02)	1.00 (0.88–1.12)	0.99 (0.88–1.12)	0.97 (0.86–1.09)	0.98 (0.87–1.11)	0.94 (0.83–1.06)
**Total fatigue**								
No	734	3.97	ref	ref	ref	ref	ref	ref
Yes	464	5.52	**1.58 (1.35**–**1.84)**	**1.60 (1.37**–**1.87)**	**1.60 (1.38**–**1.87)**	**1.50 (1.28**–**1.76)**	**1.44 (1.22**–**1.68)**	**1.24 (1.04-1.41)**

Model 2: adjusted for demographic factors (age at survey, sex, education).

Model 3: model 2 + clinical factors (years since diagnosis, cancer type).

Model 4: model 3 + comorbidity.

Model 5: model 4 + lifestyle factors (body mass index, smoking, physical activity).

Model 6: model 5 + psychological factors (depressive symptoms, fear of progression).

*HR* hazard ratio, *CI* confidence interval.

The bold values indicate significant results.

### Sensitivity analyses

After exclusion of survivors with active disease at the time of survey, the mortality risks in the short and long periods of follow-up remained comparable to that using the whole sample (Supplementary Table 3).

## Discussion

Affective, cognitive, physical, and total fatigue are prevalent among survivors of breast, prostate, and colorectal cancer 5–16 years post-diagnosis, affecting approximately a third of this sample. Cancer survivors were more likely to be fatigued than age- and sex-matched non-cancer controls and fatigue levels differed by age, education, sex, cancer type, employment status, and disease status. Domains of fatigue and sleep problems were associated differentially with demographic, clinical, comorbidity, lifestyle, and psychological factors, giving support to the multiple symptom concept of fatigue. The relationship between distinct symptoms of fatigue and mortality risks varies, depending on the duration of follow-up.

In a registry-based study of survivors of breast, cervical and colorectal cancer 15 years post-diagnosis, a greater proportion of survivors reported general (range 45–51%) and mental (range 32-40%) fatigue compared with non-cancer controls (42% and 32% respectively) [36]. Similarly, in our study, survivors of breast and colorectal cancers reported higher levels of affective and total fatigue scores in comparison to non-cancer controls. We found that age was associated with different domains of fatigue. Younger survivors ( < 60 years at survey) were more likely to report affective fatigue, whereas survivors aged 80 years or older were more likely to experience physical fatigue. Although these results are intuitive, they are partially in contrast to a study that found no associations between age and physical fatigue among breast cancer survivors [5]. A possible explanation for this discrepancy was that the sample of breast cancer survivors in the former study was significantly younger than that of our sample. In addition to age, other potential predisposing factors associated with the fatigue were lower education and female gender. These findings align with other studies on cancer and non-cancer populations [6, 13, 37].

Our study showed that clinical factors were not significantly associated with the domains of fatigue in our sample of long-term cancer survivors, with the exception of having undergone chemotherapy and the presence of active disease. We found that chemotherapy was associated with higher odds for experiencing cognitive fatigue, 5–15 years after diagnosis. A previous study on disease-free breast cancer survivors reported that while chemotherapy was associated with cognitive fatigue during the treatment phase, this association was no longer significant at the follow-up (median 6.3 years post-diagnosis) [13]. It is possible that some survivors in our sample could have had recent chemotherapy prior to survey due to disease progression. We also found that having active disease was associated with significantly higher odds for affective, cognitive, physical, and total fatigue. A previous study showed that survivors with advanced cancer report higher levels of general, physical, and mental fatigue compared with disease-free cancer survivors [16].

Comorbidity was associated with higher odds in all symptoms of fatigue and total fatigue. In particular, comorbid cardiovascular conditions were associated with higher odds for cognitive, physical and total fatigue, and inflammation and skeletal comorbidity increased the odds for physical and total fatigue. Of note, multimorbidity more than doubled the odds in all symptoms of fatigue and total fatigue. In a previous large population-based cohort study, multimorbidity was an important driver of severe and chronic fatigue in non-cancer populations [37]. In that study, a dose-response relationship was noted between fatigue severity and having an increasing number of chronic diseases. Additionally, in our study, having ever been diagnosed with depression, having depressive symptoms, and fear of progression were associated with all symptoms of fatigue and with sleep problems, with highest odds noted for affective, physical and total fatigue. These results are congruent with other studies that included long-term survivors of breast and colorectal cancer, where psychological factors were associated with mental and general fatigue [17, 36]. As with other studies [13, 17], our results indicate that lifestyle factors, including smoking, being overweight or obese, and insufficient physical activity were associated with fatigue symptoms, with the highest odds observed for physical and total fatigue.

Our findings suggest that fatigue is an independent risk factor for mortality, corresponding with previous studies on cancer and non-cancer populations [20, 38, 39]. Furthermore, it was observed that the relationship between fatigue and mortality was not consistent across the domains and was contingent on the duration of follow-up. In the shorter period of follow-up (5 years post-survey), an elevated mortality risk remained for affective (18%), physical (66%), and total (45%) fatigue after adjustment for comorbidity, lifestyle, and psychological factors. These results are in line with population-based studies involving older adults that showed perceived physical fatigue increased the risk of death by 2.3-fold [40], and that the risk differed by comorbid chronic condition [41]. In addition to physical fatigue, we observed that affective fatigue was an independent risk factor for mortality. A previous study with breast cancer survivors showed that psychological distress and anxiety were independent predictors of recurrence-free and overall survival [21]. In contrast to the shorter follow-up period, cognitive and total fatigue remained significantly associated with 20% and 24% increased mortality in the longer period of follow-up ( ≥ 6 years after survey), respectively. The association between cognitive fatigue and mortality in the longer follow-up period is intriguing. We speculate on the role of persisting inflammation in this association. A study reported that disease-free breast cancer survivors who were treated with chemotherapy 20 years previously had higher levels of inflammatory markers and lower general cognitive performance in comparison with non-cancer controls [42]. In another study of survivors with localized colorectal cancer, baseline inflammatory markers (collected pre-chemotherapy), fatigue, and cognitive function were not associated with five-year survival [43]. Further studies with longer follow-up and more detailed assessments of treatments are needed to explore the association between inflammation, cognitive fatigue and mortality.

The association between fatigue and mortality was contingent to the duration of follow-up. We observed differences in the characteristics of participants, which could explain this difference. Participants who died in the shorter follow-up period were more commonly diagnosed for a longer period before the survey, treated with hormone therapy, were former or current smokers, and had a higher fear of progression, than those who died in the longer follow-up period (Supplementary Table 2). However, in both periods of follow-up, participants who died were more likely to be older at the time of survey ( ≥ 70 years), male colorectal cancer or prostate cancer survivors, not in a partnered relationship, lower educated, obese, or not physically active (Supplementary Table 2). They were also more likely to have multimorbidity or depressive symptoms (Supplementary Table 2). Our finding that male participants with fatigue were more likely to have higher mortality risks than female fatigued survivors is in line with a previous study that found a significant association between fatigue and higher mortality in male colorectal cancer survivors, but not in female survivors of colorectal  or endometrial cancer [20].

### Clinical implications

Cancer and its treatments can accelerate the aging process and increase the risk for new morbidity and poorer health outcomes in comparison to non-cancer individuals of similar age [14, 26, 44]. These increased risks are particularly relevant since a large proportion of cancer survivors are above 60 years of age [45]. Effects such as fatigue or frailty could be attributed to advancing age and potentially dismissed as not cancer-related in older cancer survivors [46]. In our study, physical fatigue and comorbidity were associated with mortality. Previous studies have indicated that cancer survivors with multimorbidity are likely to have fragmented care across medical specialties and have an increased risk for polypharmacy which may contribute to greater symptom burden [47, 48]. Oncology specialists may prefer other specialists in primary and secondary care to manage non-cancer related conditions [49]. Therefore, it is imperative that there is better coordination and integration between the medical specialists who provide care for survivors with multimorbidity [47].

We found that psychological factors were associated with affective fatigue and with mortality. A previous study showed that anxiety and depression increased the risk of mortality among cognitively intact nursing home residents, independent of their cancer diagnosis [50]. There is ample evidence indicating that female survivors report higher levels of psychological distress than male survivors [51, 52]. While levels of psychological distress among female cancer survivors tend to remain stable, distress levels may increase among male survivors due to increasing physical problems [53]. In our study, male survivors were more likely to die than female survivors (60% versus 40%) during the follow-up (Supplementary Table 2). Of note, unlike physical fatigue which could be visually apparent, affective fatigue may be underestimated by healthcare providers [54]. Taken together, these findings indicate that healthcare providers should monitor cancer survivors for psychological distress, particularly older male survivors without a partner, and refer them to support networks or psychological care, where necessary.

The symptoms of fatigue and mortality were associated with different variables. This suggests that interventions for fatigue may not be a ‘one size fits all’ [55]. Therefore, screening for fatigue should incorporate not only the different fatigue symptoms but also include assessment of the survivors’ characteristics and situation. Fatigue management needs to be personalized to the needs of cancer survivors [55, 56]. Encouraging a more physically active life during survivorship may reduce physical fatigue and cognitive fatigue [57], and could reduce mortality risks in older persons with frailty/pre-frailty or cognitive impairment [58]. Survivors who report having high levels of distress or sleep problems may benefit more from cognitive-behavioral therapy or mind-body therapies [59].

### Strengths and limitations

Our population-based study investigated an understudied topic, the fatigue experience of cancer survivors 5–16 years post-diagnosis and its prognostic implications up to 25 years post-diagnosis. We compared the prevalence and mean fatigue scores with a non-cancer control group. In addition, the sample size was adequate to allow for subgroup analyses. Nevertheless, we acknowledge that with a response rate of 41%, our sample may not be representative of all long-term cancer survivors. In addition, our findings may be generalizable to survivors who have survived at least five years since cancer diagnosis. There is a possibility of underestimation of results as survivors with poorer health status or higher levels of fatigue may be less likely to participate in our study [60]. We used the 75^th^ percentile scores of the adult norm population as an indicator of fatigue, which could be arbitrary. Nevertheless, we found that fatigue remained an independent risk factor for mortality despite adjustment for an extensive range of covariates. However, residual confounding cannot be ruled out. We have information on comorbid conditions and disease status at time of survey but it is possible that new comorbid conditions or a new cancer is diagnosed during follow-up, which could increase the risk of mortality.

## Conclusions

Our study demonstrated that affective, cognitive, and physical fatigue is prevalent in a sample of cancer survivors, 5–16 years after diagnosis. The results support an understanding that fatigue comprises multiple symptoms. Multimorbidity was found to be an important factor associated with all symptoms of fatigue. The association between the symptoms of fatigue and mortality remained significant, albeit attenuated after adjusting for lifestyle, comorbidity, and psychological factors. These results emphasize the clinical relevance of screening for different symptoms of fatigue and in the clinical management of multimorbidity in long-term cancer survivors. Lowering the burden of fatigue in cancer survivors by a comprehensive approach might result in better survival.

## Supplementary information

Supplementary Tables and Figures

Strobe checklist

## Data Availability

Data is available from the authors upon reasonable request.
