# Supplementary material for: Fatigue in long-term cancer survivors: prevalence, associated factors, and mortality. A prospective population-based study
Source: Br J Cancer. 2025 Jul 15;133(6):831–43. doi: 10.1038/s41416-025-03116-z (PMC12449452; doi:10.1038/s41416-025-03116-z)
Supplement: Supplementary file 1 — Supplementary Tables and Figures [file 41416_2025_3116_MOESM1_ESM.docx]

**Title: Fatigue in long-term cancer survivors: prevalence, associated factors, and mortality. A prospective population-based study**

**Authors:** **Melissa S.Y. Thong^1^, Daniela Doege^1^, Lena Koch-Gallenkamp^2^, Heike Bertram^3^, Andrea Eberle^4^, Bernd Holleczek^5^, Alice Nenneke^6^, Annika Waldmann^7^, Sylke Ruth Zeissig^8,9^, Ron Pritzkuleit^10^, Elmar Brähler^11,12^, Hermann Brenner^2,13^, Volker Arndt^1^**

^1^ Cancer Survivorship, Division of Clinical Epidemiology and Aging Research, German Cancer Research Center (DKFZ), Heidelberg, Germany

^2^ Division of Clinical Epidemiology and Aging Research, DKFZ, Heidelberg, Germany

^3^ Cancer Registry of North Rhine-Westphalia, Bochum, Germany

^4^ Bremen Cancer Registry, Leibniz Institute for Prevention Research and Epidemiology - BIPS, Bremen, Germany

^5^ Saarland Cancer Registry, Saarbrücken, Germany

^6^ Hamburg Cancer Registry, Hamburg, Germany

^7^ Institute for Social Medicine and Epidemiology, University of Lübeck, Lübeck, Germany

^8^ Institute of Clinical Epidemiology and Biometry (ICE-B), Julius Maximilian University of Würzburg, Würzburg, Germany

^9^ Cancer Registry of Rhineland-Palatinate, Mainz, Germany

^10^ Cancer Registry of Schleswig-Holstein, Lübeck, Germany

^11^ Department of Medical Psychology and Medical Sociology, University Hospital Leipzig, Leipzig, Germany

^12^ Department of Psychosomatic Medicine and Psychotherapy, University Hospital Medical Center of the Johannes Gutenberg University Mainz, Mainz, Germany

^13^ German Cancer Consortium (DKTK), DKFZ, Heidelberg, Germany


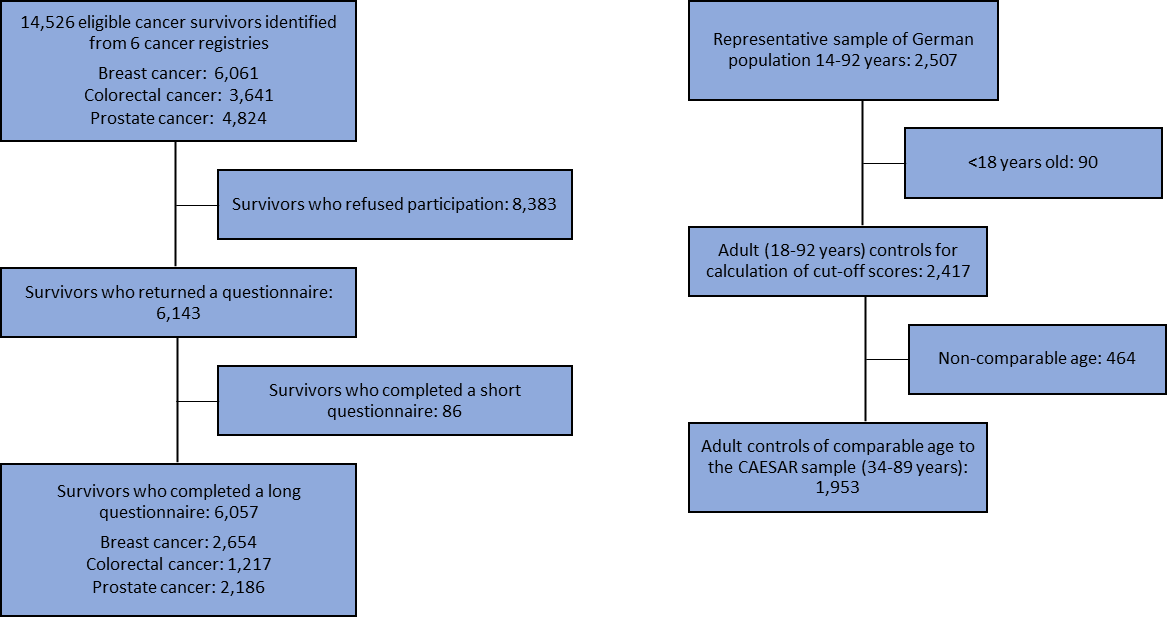


**Supplementary Figure 1. Flowchart of inclusion of cancer survivors and non-cancer controls**


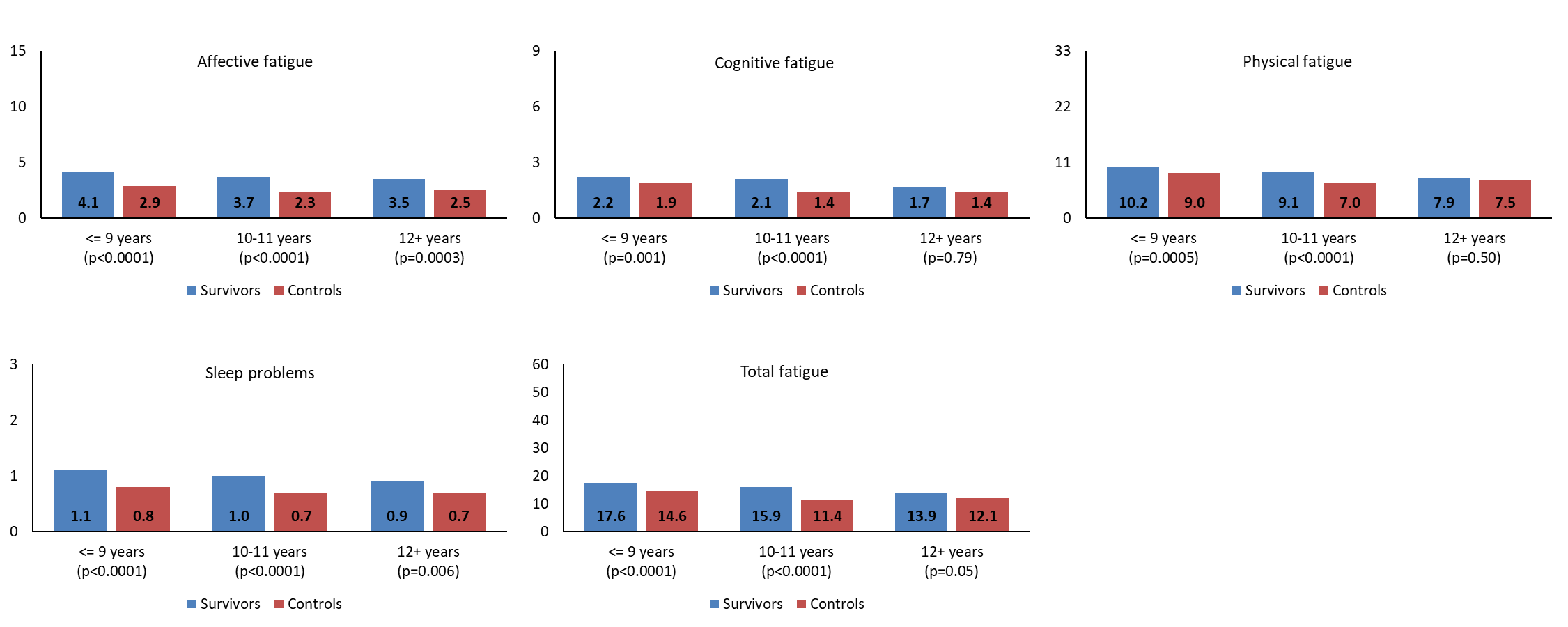


**Supplementary Figure 2.** **Mean fatigue scores of cancer survivors and non-cancer controls, stratified by education.**

Models are adjusted for age at survey and sex. The y-axis indicates the maximum score range and the mean values are shown in the columns.

All results are based on 25 imputations of missing values.


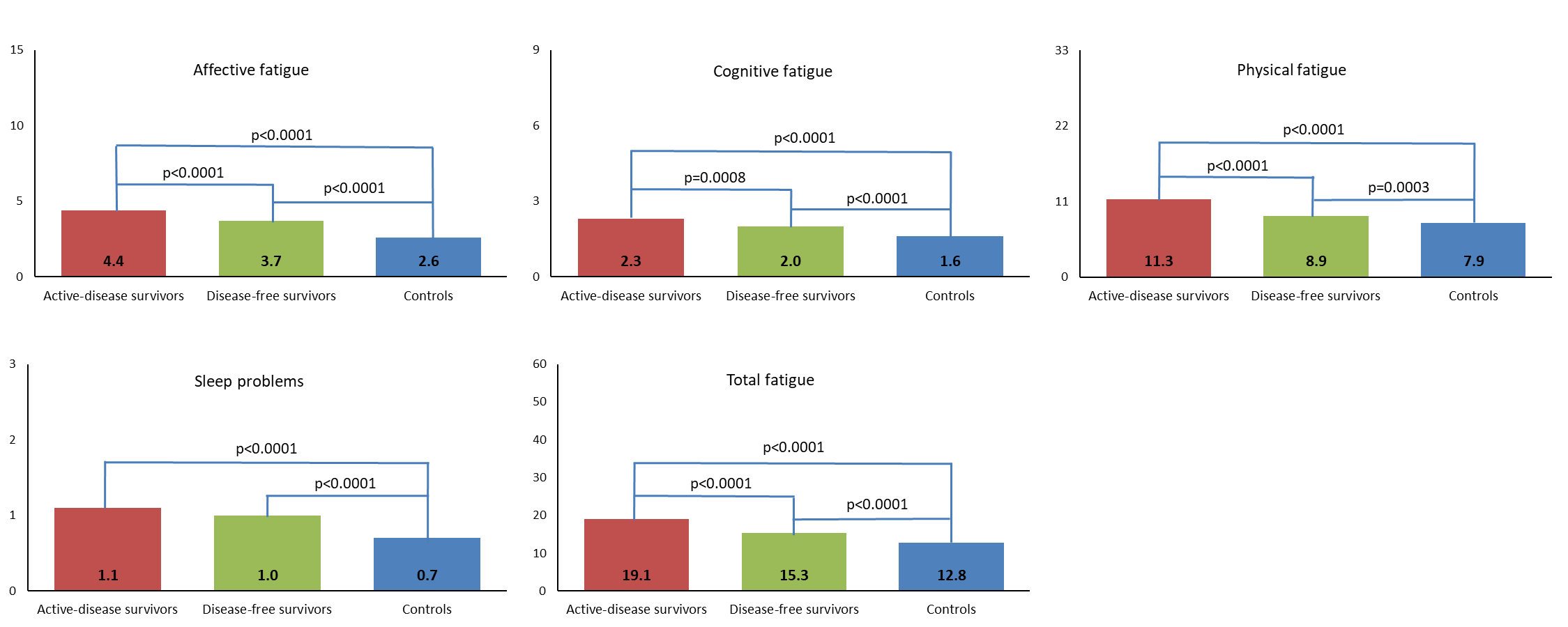


**Supplementary Figure 3.** **Mean fatigue scores of cancer survivors (with active disease or disease-free) and controls.**

Models are adjusted for age at survey, sex, and education. Disease-free: stage I-III at diagnosis and remaining disease-free at time of survey according to self-report; active disease: stage IV at diagnosis or subsequent recurrence, metastasis, or second primary cancer. The y-axis indicates the maximum score range and the mean values are shown in the columns.

All results are based on 25 imputations of missing values.


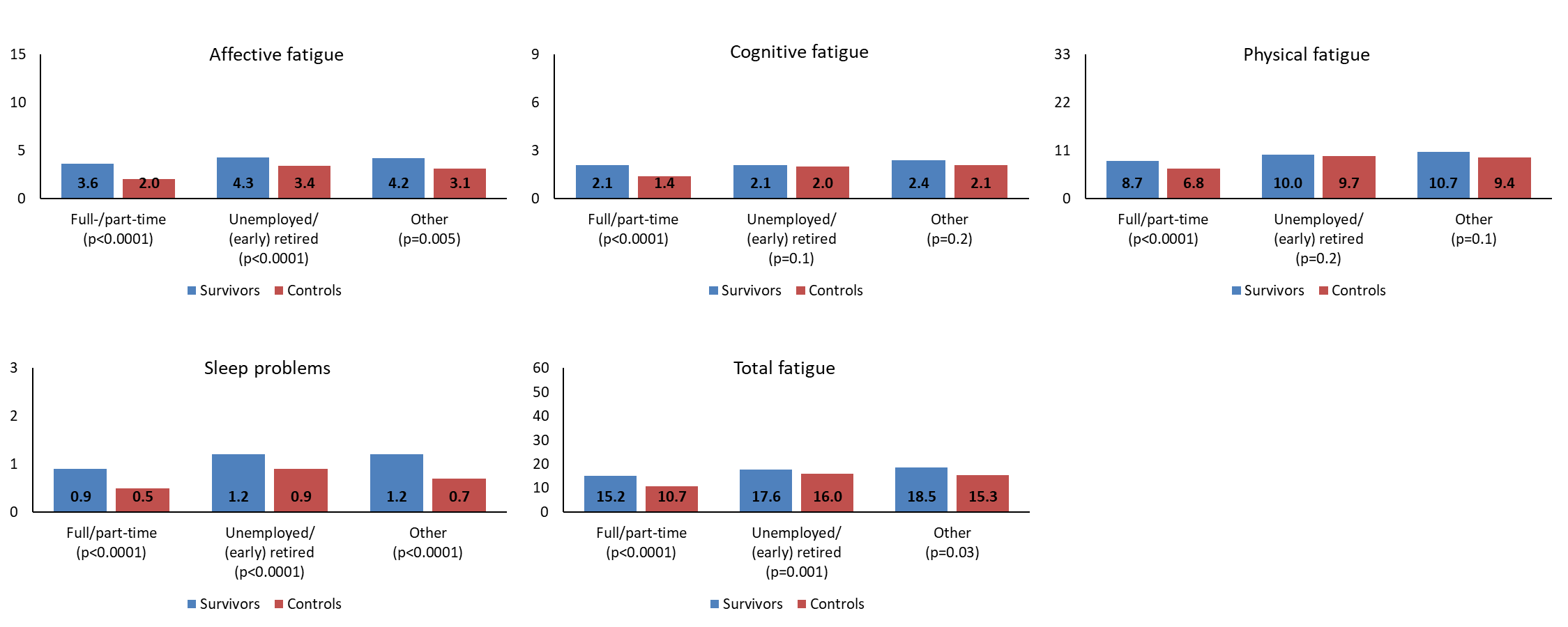


**Supplementary Figure 4.** **Mean fatigue scores of cancer survivors and non-cancer controls, stratified by employment status.**

Models are adjusted for age at survey and sex. The y-axis indicates the maximum score range and the mean values are shown in the columns.

All results are based on 25 imputations of missing values.

**Supplementary Table 1. Prevalence of fatigue in cancer survivors (n=6057) and population controls (n=1953)**

| % | **Affective fatigue** | | **Cognitive fatigue** | | **Physical fatigue** | | **Sleep problems** | | **Total fatigue** | | |
| --- | --- | --- | --- | --- | --- | --- | --- | --- | --- | --- | --- |
|  | **Survivors** | **Controls** | **Survivors** | **Controls** | **Survivors** | **Controls** | **Survivors** | **Controls** | **Survivors** | **Controls** | |
| **Total** | 39 | 33 | 35 | 28 | 34 | 28 | 63 | 47 | 35 | 28 | |
|  |  |  |  |  |  |  |  |  |  |  | |
| **Age, years** | | | | | | | | | | |  |
| <60 | 55 | 33 | 40 | 24 | 36 | 23 | 68 | 44 | 42 | 24 | |
| 60-69 | 39 | 30 | 32 | 31 | 28 | 29 | 65 | 50 | 31 | 29 | |
| 70-79 | 35 | 37 | 34 | 37 | 34 | 39 | 60 | 56 | 34 | 36 | |
| ≥80 | 35 | 34 | 41 | 51 | 46 | 53 | 63 | 68 | 42 | 47 | |
|  |  |  |  |  |  |  |  |  |  |  | |
| **Sex** | | | | | | | | | | |  |
| Male | 32 | 30 | 35 | 26 | 32 | 25 | 55 | 45 | 32 | 25 | |
| Female | 45 | 35 | 35 | 30 | 35 | 30 | 71 | 49 | 38 | 31 | |
|  |  |  |  |  |  |  |  |  |  |  | |
| **Education, years** | | | | | | | | | | |  |
| <= 9 | 41 | 34 | 39 | 33 | 39 | 32 | 65 | 50 | 40 | 32 | |
| 10-11 | 38 | 33 | 33 | 23 | 31 | 22 | 63 | 44 | 33 | 23 | |
| ≥12 | 35 | 30 | 29 | 23 | 25 | 23 | 58 | 44 | 28 | 25 | |

**Supplementary Table 2. Characteristics of survivors according to vital status and follow-up**

| % | **Short follow-up**  **(up to 5 years after survey)** | | | **Longer follow-up ***  **(≥6 years after survey)** | | |
| --- | --- | --- | --- | --- | --- | --- |
|  | **Alive (n=5240)** | **Died (n=817)** | **p-value** | **Alive**  **(n=3944)** | **Died**  **(n=1198)** | **p-value** |
| **Sex** |  |  | **<0.0001** |  |  | **<0.0001** |
| Male | 46 | 60 |  | 42 | 60 |  |
| Female | 54 | 40 |  | 58 | 40 |  |
| **Sex by cancer type** |  |  | **<0.0001** |  |  | **<0.0001** |
| Female, breast | 46 | 33 |  | 49 | 33 |  |
| Female, CRC | 9 | 7 |  | 9 | 7 |  |
| Male, CRC | 11 | 15 |  | 11 | 12 |  |
| Male, prostate | 35 | 45 |  | 31 | 48 |  |
| **Age at survey, years** |  |  | **<0.0001** |  |  | **<0.0001** |
| <60 | 17 | 7 |  | 20 | 6 |  |
| 60-69 | 31 | 19 |  | 35 | 19 |  |
| 70-79 | 44 | 50 |  | 41 | 29 |  |
| 80+ | 8 | 25 |  | 4 | 20 |  |
| **Education, years** |  |  | **0.0006** |  |  | **<0.0001** |
| <=9 | 52 | 59 |  | 50 | 59 |  |
| 10-11 | 24 | 21 |  | 26 | 19 |  |
| 12+ | 23 | 20 |  | 25 | 20 |  |
| **In a partnered relationship** |  |  | **0.03** |  |  | **0.003** |
| No | 25 | 29 |  | 24 | 28 |  |
| Yes | 75 | 71 |  | 76 | 72 |  |
| **Time since diagnosis, years** |  |  | **0.003** |  |  | 0.2 |
| <10 | 81 | 76 |  | 82 | 80 |  |
| 10+ | 19 | 24 |  | 18 | 20 |  |
| **Cancer stage** |  |  | **<0.0001** |  |  | **<0.0001** |
| I | 30 | 19 |  | 32 | 23 |  |
| II | 47 | 46 |  | 47 | 46 |  |
| III | 21 | 27 |  | 19 | 27 |  |
| IV | 3 | 8 |  | 3 | 5 |  |
| **Chemotherapy** |  |  | 0.2 |  |  | **<0.0001** |
| Yes | 39 | 41 |  | 41 | 32 |  |
| No | 61 | 59 |  | 59 | 68 |  |
| **Radiotherapy** |  |  | 0.8 |  |  | 0.5 |
| Yes | 57 | 57 |  | 57 | 56 |  |
| No | 43 | 43 |  | 43 | 44 |  |
| **Hormone therapy** |  |  | **0.01** |  |  | 0.07 |
| Yes | 38 | 43 |  | 39 | 35 |  |
| No | 62 | 57 |  | 61 | 65 |  |
| **Disease progression** |  |  | **<0.0001** |  |  | **<0.0001** |
| Yes | 8 | 19 |  | 7 | 11 |  |
| No | 92 | 81 |  | 93 | 89 |  |
| **Self-reported comorbidity** |  |  | **<0.0001** |  |  | **<0.0001** |
| None | 40 | 28 |  | 42 | 34 |  |
| Cardiovascular disease | 8 | 13 |  | 7 | 10 |  |
| Skeletal | 5 | 6 |  | 4 | 7 |  |
| Inflammation | 17 | 12 |  | 18 | 14 |  |
| Depression | 6 | 5 |  | 7 | 4 |  |
| Multimorbidity | 25 | 35 |  | 22 | 32 |  |
| **Body mass index** |  |  | **0.03** |  |  | **<0.0001** |
| <25 | 32 | 31 |  | 33 | 27 |  |
| 25 - <30 | 47 | 44 |  | 48 | 47 |  |

**Supplementary Table 2. continued**

| % | **Short follow-up**  **(up to 5 years after survey)** | | | **Longer follow-up ***  **(≥6 years after survey)** | | |
| --- | --- | --- | --- | --- | --- | --- |
|  | **Alive (n=5240)** | **Died (n=817)** | **p-value** | **Alive**  **(n=3944)** | **Died**  **(n=1198)** | **p-value** |
| ≥30 | 21 | 25 |  | 19 | 26 |  |
| **Smoking** |  |  | **0.008** |  |  | 0.2 |
| Never | 80 | 75 |  | 80 | 78 |  |
| Former | 10 | 13 |  | 10 | 11 |  |
| Current | 10 | 12 |  | 10 | 11 |  |
| **MVPA 150 minutes/ week** |  |  | **<0.0001** |  |  | **<0.0001** |
| No | 56 | 79 |  | 53 | 64 |  |
| Yes | 44 | 30 |  | 47 | 36 |  |
| **Geriatric Depression Scale** |  |  | **<0.0001** |  |  | **<0.0001** |
| Asymptomatic | 78 | 59 |  | 80 | 72 |  |
| Symptomatic | 22 | 41 |  | 20 | 28 |  |
| **Fear of progression** |  |  | **<0.0001** |  |  | 0.09 |
| No | 88 | 82 |  | 88 | 87 |  |
| Yes | 12 | 18 |  | 12 | 13 |  |

*98 participants were censored in the longer period of follow-up

Percentages may not add up to 100% due to rounding off

**Supplementary Table 3. Risk estimates of fatigue on mortality in disease-free cancer survivors**

|  | **Short follow-up (up to 5 years after survey)** | | | **Long follow-up (6 years or more after survey)** | | |
| --- | --- | --- | --- | --- | --- | --- |
|  | **Total**  **Deaths n=657** | **Crude mortality rate**  **(100/person-years)** | **HR_adjusted_ (95% CI)** | **Total**  **Deaths**  **n=1064** | **Crude mortality rate (100/person-years)** | **HR_adjusted_ (95%CI)** |
| **Affective Fatigue** |  |  |  |  |  |  |
| No | 350 | 2.20 | ref | 677 | 4.28 | ref |
| Yes | 307 | 3.19 | **1.24 (1.03-1.48)** | 387 | 4.25 | 1.08 (0.90-1.29) |
| **Cognitive Fatigue** |  |  |  |  |  |  |
| No | 378 | 2.23 | ref | 635 | 3.75 | ref |
| Yes | 280 | 3.25 | 1.00 (0.84-1.19) | 429 | 5.39 | **1.26 (1.09-1.45)** |
| **Physical fatigue** |  |  |  |  |  |  |
| No | 323 | 1.84 | ref | 669 | 3.75 | ref |
| Yes | 334 | 4.20 | **1.60 (1.34-1.93)** | 395 | 5.59 | 1.20 (1.00-1.44) |
| **Sleep problems** |  |  |  |  |  |  |
| No | 230 | 2.39 | ref | 422 | 4.52 | ref |
| Yes | 428 | 2.69 | 1.07 (0.90-1.26) | 642 | 4.12 | 0.94 (0.82-1.07) |
| **Total fatigue** |  |  |  |  |  |  |
| No | 332 | 1.95 | ref | 656 | 3.80 | ref |
| Yes | 325 | 3.83 | **1.42 (1.18-1.72)** | 409 | 5.34 | **1.33 (1.11-1.60)** |

HR: hazards ratio; CI: confidence interval

Models are adjusted for age at survey, sex, education, years since diagnosis, cancer type, comorbidity, body mass index, smoking, physical activity, depressive symptoms, fear of progression
